# The Microscope in Medicine

**Published:** 1891-05-09

**Authors:** Frank J. Wethered


### THE MICROSCOPE IN MEDICINE.

By Frank J. Wethered, M.D.

### XIV.—THE EXAMINATION OF VOMIT, FÆCES, AND MORBID FLUIDS.

The examination of Vomit is more important as regards its chemistry than from the information which may be gained with the help of the microscope. Chemical tests, indeed, when applied to vomited matters, are of the highest importance, indicating the presence of poisons, &c. The microscopic tests for these bodies are less reliable, and therefore will not be considered here. The vomited matters ought to be examined as soon as possible after ejection, as many substances present undergo rapid alteration. The more solid portions should be separated by filtration through muslin, and the filtrate allowed to stand for a few hours to allow the deposit to settle.

In the mass left in the filter various food matters will be found, such as vegetable fibres, shreds of muscular tissue, fat globules, starch granules, &c., and unless the observer makes himself familiar with these, great errors may arise. Occasionally, too, pieces of croupous or diphtheritic membranes may be found, having been dislodged during the act of vomiting; these are best examined by leaving them out in water.

In very rare cases, also, shreds of gastric mucous membrane, separated in the process of acute destructive inflammation. sediment may be removed by means of a pipette to glass slides for microscopic examination.

The filtrate as just stated should be allowed to stand preferably in a conical glass, and after some time portions of the

Large quantities of undigested food stuff will of course be seen, but the most important bodies to search for are torulæ and sarcinæ.

*Torulæ* are occasionally present in considerable numbers. They appear as rounded cells $\frac{1}{3000}$ of an inch in diameter, they are usually arranged in chains and exhibit lateral buds. They are found in cases of obstructive disease of the pylorus.

In connection with the same condition *Sarcinæ* are found. These bodies have a very characteristic form, appearing like small bales of goods. They are rather larger than red blood corpuscles, and have a silver-grey colour. When a solution of iodine is added they assume a deep mahogany brown.

*Red blood corpuscles* appear in the vomit when there is carcinoma or ulcer of the stomach; owing to the action of the digestive juices their appearance is often greatly altered so that they can scarcely be recognised.

*Epithelial cells* occur in variable numbers, squamous cells from the mouth and cylindrical from the mucous membrane of the stomach. No definite conclusions can be drawn from their presence. It is stated by some authors that "cancer cells" are found when there is carcinoma of the stomach, but it is very doubtful whether such cells can be detected with any certainty.

In considering the microscopical examination of the Fæces, much may be repeated which has been said about vomit.

The grosser objects which have to be searched for are gall-stones, intestinal concretions (formed by the accumulation of indigestible articles), and mucous and membranous casts. The first two do not require further notice. With regard to the casts, these are chiefly met with in cases of membranous colitis. They consist of a fibrinous material, enclosing a large number of epithelial cells, and in some cases have, exhibited on their surface, pits corresponding to the mouths of the glands.

Portions of the fæces should be shaken up with water, and, after some hours, some of the deposit removed for examination in the usual way. The main objects seen will naturally be undigested remains of food, as in the case of vomit; but, in addition, other objects have occasionally to be sought for which are of the greatest importance. These are the various parasites with their ova, and certain micro-organisms.

It would be impossible to describe here all the parasites that are met with, and only the more common will be described.

The cestode most frequently seen in this country is the Tænia mediocanellata. It is divided into a head and neck and a number of segments called proglottides. The most characteristic is the head. This should always be sought for; it is square in shape, and has four suckers, usually deeply pigmented. The ova are occasionally seen; they are oval in shape, and furnished with six hooklets. For a further description of the anatomy and life history of this worm the reader is referred to the larger text-books, and the same remark applies to the other parasites that are described here.

The *Tænia Solium* is rarely met with in England. Its head differs very much from that of the worm just described. It is rounded and furnished with four suctorial discs; these are surmounted with a crown of hooklets of two sizes, large and small. Forming the apex of the head is a prominence called the "rostellum." The ova are spherical, and are also provided with hooklets.

The *Ascaris Lumbricoides* or *Round Worm* does not need the microscope for its recognition. It very much resembles an ordinary earthworm in form. The eggs are blunted oval-bodies, and yellowish-brown in colour.

The *Thread Worm* (*Oxyuris vermicularis*) is a white thread-like body, with a blunt anterior, and tapering posterior extremity. The eggs are roughly oval in shape, with granular contents.

Other rarer parasites are the Bothriocephalus latus, Distoma hepaticum, Trichocephalus dispar, &c., and Anchylostoma duodenale. These, however, are very seldom met with in this country.

Some micro-organisms are of importance.

Perhaps the most interesting is the *comma bacillus* found by Koch in the stools of patients suffering from Asiatic cholera. They may be demonstrated by making cover-glass preparations from the stools, and staining with methylene blue or fuchsine. The bacilli are curved rods about half the length of the tubercle-bacillus, sometimes joined together into wavy threads—spirilla—although this condition is more frequently seen in artificial "drop-cultures."

Various organisms have been described as causing *Cholera*

*Nostras.* Finkler and Prior have described a bacillus very much resembling Koch's, but its pathological value is doubtful. Escherich has also recently described a bacillus in connection with this disease, and from his experiments it would certainly seem that this microbe has a distinct aetiological relation with this form of "cholera."

In sections of the intestine taken from patients who have died from *Enteric Fever*, characteristic organisms are found, and they also occur in the stools during life; but as they possess no special staining properties, they cannot be distinguished from the numerous other bacteria which are always found.

*Tubercle bacilli* have been found in the fæces in patients afflicted with tubercular disease of the bowel. They are demonstrated in the usual way by Neelsen's method (see "Examination of Sputum"); a small portion of the fæces is pressed between two cover-glasses and treated in the manner already described.

THE EXAMINATION OF MORBID FLUIDS includes the microscopical investigation of deposits from serous effusions, abscesses, and cysts.

As regards *serous effusions*, the chief interest lies in the search for micro-organisms, especially the tubercle bacillus. The fluid should be allowed to stand for twenty-four hours, and some of the deposit poured out on to a black dish, and if any opaque particles be seen they should be selected for investigation, cover-glass preparations being made and stained by Neelsen's method. In this way the writer has succeeded in finding tubercle bacilli in perfectly clear serum.

In the examination of pus from abscesses again the search for micro-organisms is important. Tubercle bacilli may be found, or actinomyces. In regard to the last named, it is to be urged that all purulent discharges from the chest, when there is any doubt as to their nature, should be investigated. If the "ray-fungus" be present, the clubs will be found in unstained specimens, whilst the mycelial threads may be brought into view by Gram's method of staining.

In addition to micro-organisms in the sediment of purulent fluid may occasionally be found crystals of triple phosphates, cholesterine, fatty acids, and hæmatoidi, also echinococcus hooklets. The form of CYST yielding most information on microscopic examination is the *hydatid* cyst. If the deposit from such a cyst be examined, the chracteristic "scolices" will be found. These are oval in form, and are furnished with a crown of hooklets and four suckers. They are generally studded with bile-stained calcareous granules. Detached hooklets are more commonly seen, and are sometimes the only evidence of the nature of the cyst. In addition, fragments of the "cuticula" or outer coat of the cyst wall are occasionally seen.

The microscopic examination of other forms of cysts lends little aid to diagnosis.

